# Small Cell Neuroendocrine Carcinoma of the Cervix in Pregnancy: A Case Report and Review

**DOI:** 10.1155/2019/8028459

**Published:** 2019-04-01

**Authors:** Linglan Pan, Renyan Liu, Xiujie Sheng, Dunjin Chen

**Affiliations:** ^1^The Institute of Gynecology and Obstetrics, The Third Affiliated Hospital of Guangzhou Medical University, Guangzhou, Guangdong 510150, China; ^2^Department of Pharmacology, Upstate Medical University, State University of New York, Syracuse, New York 13210, USA

## Abstract

Small cell neuroendocrine carcinoma of the cervix is a rare subtype of cervical cancer. Here we report a case in which a 27-year-old female patient presented at 34-week gestation with abnormal vaginal bleeding, underwent normal labor, and gave birth to a healthy neonate. Her pregnancy was complicated with a cervical tumor which turned out to be small cell neuroendocrine cervical carcinoma. We reviewed and discussed the features, diagnosis, and prognosis of small cell neuroendocrine carcinoma of the cervix.

## 1. Introduction

Small cell neuroendocrine carcinoma of the cervix (SCNCC) is a high-grade malignant tumor. It rarely occurs during pregnancy and only 18 cases have been reported so far. Diagnosis at early FIGO (International Federation of Gynecology and Obstetrics) stage is of paramount importance for a better prognosis of patients with such cancer.

## 2. Presentation of Case

**Figure 1:**
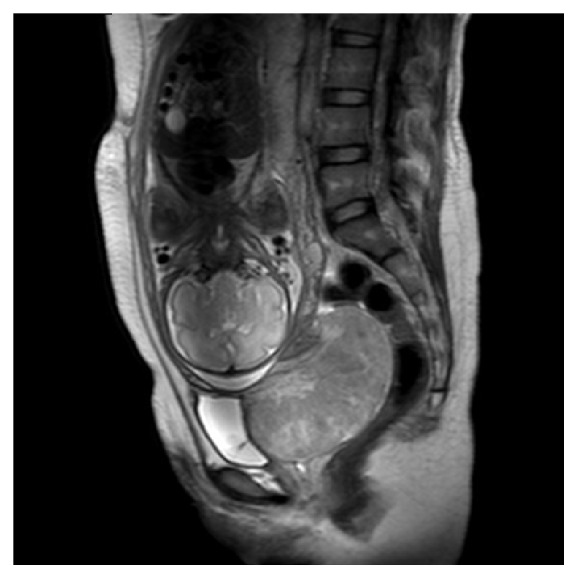
Pelvic MRI.

A 27-year-old primigravida woman presented with intermittent vaginal spotting and abdominal pain occasionally for more than one month at 34 weeks of gestation in September 2014. In the past month, she just had bed rest at home for preventing preterm birth. When she was in the emergency room at first time, a large tumor about 10cm in diameter was found in her cervix by pelvic examination. No abnormal findings were found before or early in this pregnancy. However, the patient refused HPV (human papillomavirus) testing when she was bleeding, let alone biopsy. The ultrasound revealed a myoma in her cervix. Cervical myoma was diagnosed and she received hemostasis and miscarriage prevention. When she was in the emergency room at second time for the reason of recurrent vaginal bleeding, the tumor was friable and bled by speculum examination, without typical cervix. The pelvic magnetic resonance image (MRI) was arranged for her immediately and it showed that the patient's cervical canal was obviously expanded, with a huge cauliflower-shape tumor (9.6-cm X 10.0-cm). There was slightly high signal in T2W, heterogeneous enhancement in arterial phase, persistent enhancement in delayed phase, high signal presented in DWI, and reduced ADC (Figure 1). Since this lesion was big, the patient was admitted to hospital. After admission, the patient's vaginal bleeding volume was larger than her typical menstruation and she had regular uterine contractions. An acute hemorrhage prompted an emergency cesarean section with delivery of viable male infant with weight of 1980g. Because of the potential problem of hemorrhage, a notorious complication of myomectomy performed at caesarean section, the obstetrician did not treat the cervical tumor at the cesarean section.

**Figure 2:**
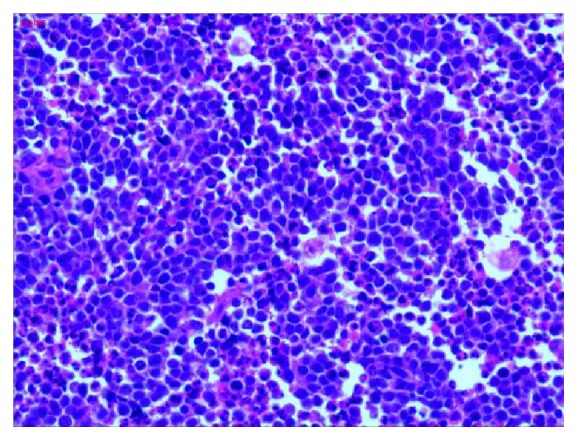
Histology of the cervical small cell carcinoma (X200). There was hypercellularity and scanty stroma. The cells were arranged in ribbons, lines, or waves, which are typical of small cell carcinomas.

**Figure 3:**
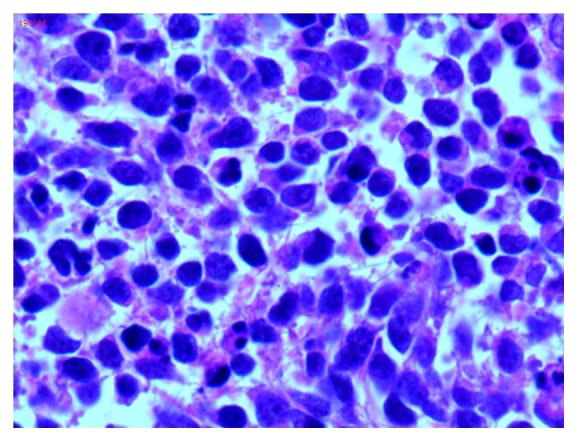
Histology of the cervical small cell carcinoma (X400). The H&E staining of the tissue section showed hypercellularity and the majority of the tumor cells were small and hyperchromatic and featured high nuclear cytoplasmic ratio.

**Figure 4:**
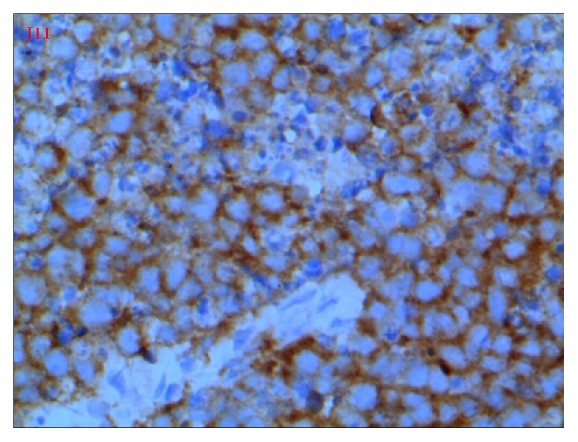
X400, CD56, positive.

**Figure 5:**
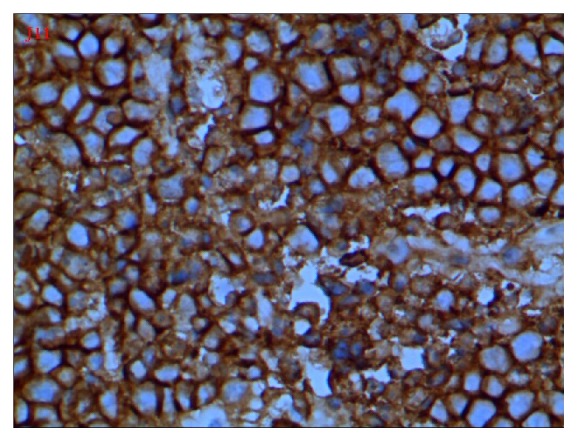
X400, Syn, positive.

Following cesarean section, her lochia was normal and she would have undergone myomectomy after puerperium. However, about 20 days later, her vaginal bleeding suddenly became worse along with severe lower abdominal pain and she was admitted again. Pulmonary CT and ultrasound of upper abdomen were negative for cancer metastasis. The tumor markers such as CA125 and CA199 were normal. A pelvic examination revealed a 20.0-cm fragile cervical tumor, assumed to be a myoma. Soon after, the patient was treated by laparotomy. In the posterior wall of the cervix there was a big tumor (20.0-cm X 20.0-cm X 20.0-cm) which was friable with necrosis and foul odor. The tumor extended to the vagina. The bilateral adnexa were normal. Pathological studies of frozen sections of the cervical mass suggested that it was a type of small cell carcinoma. The tumor was so large; hysterectomy was performed transabdominally and transvaginally. Lymphadenectomy was not performed because of large blood loss at operation. Further detailed pathological examination indicated that the cervical mass was small cell neuroendocrine carcinoma of the cervix (SCNCC), with negative surgical margins. Immunohistochemistry study showed Syn(+++), CD56(+++), CK(-), CgA(weakly positive), and Ki-67 (index 95%) (Figures 2–5).

Even though there was no histological evidence of residual disease, the patient was diagnosed as FIGO stage III, according to clinical findings during operation. She had no history of smoking, medical diseases, or family history of malignancy. She was started on combination chemotherapy with cisplatin 70 mg/m on Day 1 by intravenous infusion (iv) and etoposide 70 mg/m on Days 1–5 iv. Only four cycles were administered due to adverse effects. The patient was referred to radiotherapy, but she refused. Unfortunately, about 11 months later, she died of the tumor.

## 3. Discussion 

The incidence of small cell neuroendocrine cervical cancer is 0.99% in The Third Affiliated Hospital of Guangzhou Medical University (5 out of 503 cervical cancers from 2005 to 2015). It is as low as 0.31–3% according to literature [1, 2]. Until now 18 cases were reported about small cell neuroendocrine cervical cancer that occurred during pregnancy [3–18].  Table 1 lists the features of all the 18 cases. The average age of all patients was 26.3 years. They were diagnosed by biopsy, cervical conization, or surgery. Nine patients were dead of disease within 4 years of diagnosis. The longest survivor was for 84 months [4]. The poorest outcome was of woman who died one month after diagnosis as stage IV-B [17]. Only one patient had family history of cancer. Her first-order cousin had cervical cancer at age 22, and the maternal grandmother died of cervical cancer [14]. The youngest gestational age was just 10 weeks and the patient remained adamant about preserving the pregnancy; then she had neoadjuvant chemotherapy (NACT) of 4 cycles and delivered at 36 weeks [3]. Only one newborn out of eighteen was dead on day 27 of life [14]. Ten patients underwent NACT. It would be a better outcome for these patients who had NACT and definitive treatment at early stage.

*Clinical manifestation and diagnosis:* Most patients with small cell neuroendocrine carcinoma of the cervix present with abnormal vaginal bleeding and some have pelvic pain and pressure-like discomfort [8, 19]. Initially small cell neuroendocrine carcinomas of the cervix may be misdiagnosed as cervical myomas such as the case in our study or rapidly growing polyps in the cervix [8]. Usually, HPV18 were positive [20]; meanwhile, cytology was often negative. The probability of making a preoperative diagnosis of small cell carcinoma was only 34.2% [21]. Due to the high rate of misdiagnosis, a biopsy should be taken for the patient in our study, but she did not agree to biopsy. Immunohistochemical studies such as staining of CD56, chromogranin A, and synaptophysin are always required to reach a final diagnosis after surgery.

*Therapy:* A study showed that primary radical surgery with subsequent adjuvant chemotherapy was the preferred treatment strategy for patients with early stage SCNCC [22]. On the other hand, primary radiation therapy had a better survival outcome than primary surgery to patients with stages IB2–II SCNCC9 [23]. In other words, the selection of primary surgery was significantly related to early FIGO stage, younger age, no detectable lymph node metastasis, and smaller tumor size [23, 24]. Therefore, in this case, an appropriate therapeutic schedule should be made as early as possible right after the cesarean section. Even though there is no evidence of nodal metastasis, adjuvant platinum-based chemotherapy should be considered owing to a high risk of distant recurrence [25, 26].

Etoposide/cisplatin (EP) was the most commonly used chemotherapeutic regimen for SCNCC [27]. In advanced FIGO stage, concurrent chemoradiation (CCRT) with at least five cycles of EP (CCRT-EP5+) was associated with significantly better 5-year FFS [24]. Patients with small size tumors (≤2cm) who received neoadjuvant chemotherapy (NACT) with radical surgery showed a lower probability of distant recurrence than that in the patients who received surgery as the primary therapy [25], but the significance of the results was limited by the small size of samples. A delay of the tumor in order to maintain normal progression of gestation is contraindicated in women whose pregnancies are complicated by SCNCC [10]. Given the high aggressiveness of SCNCC, Balderston suggested that treatment should be started immediately following the accurate diagnosis. And a viable fetus should be delivered by classical cesarean section in order to avoid the lower uterine segment. In addition, they suggested that induction of systemic therapy should be started immediately after delivery of the fetus [10]. Unfortunately, the patient in our study was not diagnosed in the early stage and there was no appropriate antepartum systemic therapy for her. Patients who prefer to postpone treatment until gestational advancement in early pregnancy may receive antepartum systemic therapy followed by NACT plus fetal surveillance [8].

*Prognosis:* The overall 5-year survival for patients with SCNCCs at stages I-IIA and IIB-IV was 36.8% and 8.9%, respectively [19], which indicates the poor prognosis. FIGO stage was the only significant factor affecting CSS, and the presence of positive surgical margins was a significant factor associated with poorer FFS in patients who receive surgery as the primary treatment [24]. In this case, the patient was FIGO stage III which indicated the poor outcome. For this reason, we hope obstetricians and gynecologists learn more about the diagnosis and management of SCNCC.

## 4. Conclusion

Recurrence of vaginal bleeding should be noticeable. Cytology and HPV testing or endocervical sampling are recommended even in pregnant women. It is crucial to recognize and choose appropriate treatment for SCNCC.

## Figures and Tables

**Table 1:** Small cell neuroendocrine cervical cancer during pregnancy: literature review.

Author	Age	FIGO	Parity	GA	NACT	Treatment	Follow up	Outcome
	(years)	stage		(weeks)			(months)	
Teefey	31	IB1	G2P1	10	4*∗*EP	C/S+RH+PLND	24	NED
Wu	25	IB1	G1P0	term	2*∗*IEP	RT+PLND+6*∗*IEP	84	NED
Wang	18	IB2	G1P1	term	4*∗*EP	Radiotherapy	5	NED
Chun	27	IB1		25	3*∗*TP	C/S+RH+PLND+PALND	46	DOD
Chun	32	IIA		28	1*∗*TC	RH+PLND+PALND	48	NED
Chun	27	IB2		28	2*∗*TP	RH+PLND+PALND+4*∗*TP	60	NED
Smyth	26	IIA	G1P0	23	3*∗*AC	C/S+4*∗*EP+ Radiation		NED
Ohwada	27	IB1	G1P0	27		C/S+RH+PLND+4*∗*EP	13	NED
Leung	26	IB2	G1P0	31		C/S+CCRT+LH+BSO	14	NED
Balderston	22	IIA	G5P3	30	3*∗*EP	Radiation+4*∗*EP	66	NED
Perrin	23	IIA	G1P0	25		C/S+RH+LSO+PLND		DOD
Chang	27	IB		36		C/S+RH+PLND		DOD
Lojek	28	IIA		25		C/S+PLND+CCRT	30	DOD
Turner	26	IB	G2P1	26		C/S+RH+PLND+6*∗*VAC+2*∗*EP	9	DOD
Jacobs	25	IB		10	DDP	RH +PLND +Radiotherapy	24	DOD
Kodousek	29	IB		28		C/S+RH+PLND+EP	6	DOD
Canto	30	IV-B	G1P0	36	1*∗*EP		1	DOD
Liu	25	IV-B		32		C/S+RH+PLND	3	DOD

FIGO, International Federation of Gynecology and Obstetrics; GA, gestational age; NACT, neoadjuvant chemotherapy; EP, cisplatin+ etoposide; TP, Paclitaxel+ Cisplatin; TC, Paclitaxel+carboplatin; VAC,vincristine+doxorubicin+ cyclophosphamide; IEP, ifosfamide+ cisplatin+ etoposide; C/S, cesarean section; RH, radical hysterectomy; RT, Radical Trachelectomy; PLND, pelvic lymph node dissection; PALND, para-aortic node dissection; CCRT, chemoradiotherapy; DOD, dead of disease; NED, no evidence of disease.
